# Performance Assessment of ChatGPT-4.0 and ChatGLM Series in Traditional Chinese Medicine for Metabolic Associated Fatty Liver Disease: Comparative Study

**DOI:** 10.2196/66503

**Published:** 2025-08-25

**Authors:** Xionghui Wang, Tianxiao Zheng, Bo Liu, Zhi Pei, Kaihan Meng, Changquan Ling

**Affiliations:** 1 Department of Gastroenterology, No. 967 Hospital of PLA Joint Logistics Support Force, Dalian, China; 2 School of Traditional Chinese Medicine, Naval Medical University, No. 800, Xiangyin Road, Yangpu District, Shanghai, 200433, China, 86 02181871561; 3 Department of Traditional Chinese Medicine, First Affiliated Hospital, Naval Medical University, Shanghai, China; 4 Medical Administration Office, No. 967 Hospital of PLA Joint Logistics Support Force, Dalian, China; 5 Department of Traditional Chinese Medicine, Second Affiliated Hospital, Naval Medical University, Shanghai, China

**Keywords:** large language models, artificial intelligence, AI medical assistants, knowledge base fine-tuning, internet-enabled LLMs, traditional Chinese medicine, Chinese language models

## Abstract

**Background:**

ChatGPT-4.0 and the ChatGLM series are novel conversational large language models (LLMs). ChatGLM includes 3 versions: ChatGLM4 (with internet connectivity but no knowledge base pretraining), ChatGLM4+Knowledge base (combining internet search capabilities with knowledge base pretraining), ChatGLM3-6B (offline knowledge base pretraining but no internet connectivity). The ability of ChatGPT-4.0 and ChatGLM to apply medical knowledge in the Chinese environment has been preliminarily verified, but the potential of the 2 models for clinical assistance in traditional Chinese medicine (TCM) is still unknown.

**Objective:**

This study aims to explore the performance of ChatGPT-4.0, ChatGLM4, ChatGLM4+Knowledge base, and ChatGLM3-6B in providing AI-assisted diagnosis and treatment for metabolic dysfunction-associated fatty liver disease within a TCM clinical framework, thereby assessing their potential as TCM clinical decision support tools.

**Methods:**

This study evaluated 4 LLMs by providing them with medical records of 87 metabolic dysfunction-associated fatty liver disease cases treated with TCM and querying them about TCM treatment plans. The answering texts from 4 LLMs were evaluated using predefined scoring criteria, focusing on 3 critical dimensions: ability in syndrome differentiation and treatment principles, confusion of concepts between TCM and Western medicine, and comprehensive evaluation of question-answering texts (comprising 6 components: ability to integrate Chinese and Western medicine, ability to formulate treatment plans, health management capacity, disease monitoring ability, self-positioning awareness, and medication safety).

**Results:**

In the evaluation module of “Ability in syndrome differentiation and treatment principles,” the performance ranking of the 4 models was: (1) ChatGLM4+ Knowledge Base, (2) ChatGLM4, (3) ChatGLM3-6B, and (4) ChatGPT-4.0. Regarding the assessment of confusion between TCM and Western medicine concepts, ChatGPT-4.0 exhibited conceptual confusion in 32 out of 87 cases, while the ChatGLM series of LLMs showed no such confusion (except for ChatGLM3-6B, which had 1 instance). In the “Comprehensive evaluation of question-answering texts” module (comprising 6 components: ability to integrate Chinese and Western medicine, ability to formulate treatment plans, health management capacity, disease monitoring ability, self-positioning awareness, and medication safety), the ranking was: (1) ChatGLM4+ Knowledge Base, (2) ChatGPT-4.0, (3) ChatGLM4, and (4) ChatGLM3-6B.

**Conclusions:**

Our study results demonstrated that real-time internet connectivity played a critical role in LLM-assisted TCM diagnosis and treatment, while offline models showed significantly reduced performance in clinical decision support. Furthermore, pretraining LLMs with TCM-specific knowledge bases while maintaining internet search capabilities substantially enhanced their diagnostic and therapeutic performance in TCM applications. Importantly, general-purpose LLMs required both domain-specific medical fine-tuning and culturally sensitive adaptation to meet the rigorous standards of TCM clinical practice.

## Introduction

ChatGPT-4.0 is the latest generation chat-based artificial intelligence (AI) language model developed by OpenAI. It is part of the GPT-4 series of models designed to provide a more fluid, accurate, and human conversation experience. Compared to the previous generation ChatGPT-3.5, ChatGPT-4.0 has significant improvements in stronger language understanding, improved context management, natural answers, extensive knowledge coverage, and multilanguage support.

ChatGLM (Generative Language Model), Chinese name: Zhipu Qingyan, is an intelligent dialogue system integrated with advanced AI technology, jointly developed by Tsinghua University KEG Lab and Zhipu AI company. It is based on the powerful language model GLM-4, with natural language understanding and generation capabilities, and can communicate smoothly and naturally with users. ChatGLM can be applied to a variety of scenarios, such as customer service, entertainment, education, etc, providing questions and answers, information retrieval, and other functions. Compared with traditional rule-based chatbots, generative language model-based chatbots can provide a more smooth, natural, and diversified conversation experience, especially in terms of understanding and answering Chinese questions. The current ChatGLM models include ChatGLM4 networking, ChatGLM4 networking + knowledge base, and ChatGLM3-6B deployed locally.

In terms of clinical decision aid, ChatGPT-4.0 has the ability to be used as an aid tool for clinical decision making. The evaluation results of ChatGPT on acute ulcerative colitis are highly consistent with those of gastroenterologists [1]. ChatGPT performs well in assisting pediatricians in the diagnosis and treatment of diseases and assisting doctor-patient communication in the Chinese environment [2], indicating that ChatGPT-4.0 has high credibility in participating in the diagnosis and treatment.

ChatGLM has demonstrated a commendable ability to provide proprietary Chinese medicine recommendations tailored to a patient’s condition, making it a promising tool for TCM-assisted diagnosis and treatment [3]. The ability of ChatGPT-4.0 and ChatGLM to apply medical knowledge in Chinese environments has been preliminarily verified [3,4], but the ability of assisting diagnosis and treatment of diseases related to traditional Chinese medicine (TCM) is still unknown.

Metabolic dysfunction-associated fatty liver disease (MAFLD), a prevalent chronic hepatic disorder, poses significant health risks through its potential progression to steatohepatitis, hepatic fibrosis, and cirrhosis [5,6]. Current global therapeutic strategies lack standardization, with conventional approaches encompassing lifestyle modifications (eg, dietary adjustments [7-10] and physical activity) and pharmacological interventions, such as resmetirom [11], antidiabetic agents (glucagon-like peptide-1 agonists [12] and pioglitazone [13]), and vitamin E [14,15], which have shown efficacy in clinical trials and meta-analyses. However, challenges remain in achieving long-term disease remission [6]. TCM offers a multifaceted therapeutic paradigm for MAFLD, using herbal decoctions, acupuncture, and acupoint massage [16-18]. Recent advances have elucidated TCM’s unique advantages through multitarget regulatory mechanisms, including modulation of miRNA networks [19], ceRNA pathways [20], gut microbiota composition [21,22], enterohepatic axis dynamics [23-25], ferroptosis inhibition [26], mitochondrial autophagy activation [27], and regulation of inflammatory/oxidative stress responses [28-30]. Nevertheless, the individualized nature of TCM’s “syndrome differentiation and treatment determination” system, coupled with its complex theoretical framework, presents dual challenges for artificial intelligence: (1) overcoming cultural-linguistic barriers in semantic parsing and (2) achieving deep integration of domain-specific knowledge for dynamic clinical decision-making. Notably, no previous studies have systematically evaluated the adaptability and reliability of large language models (LLMs) in TCM diagnostic-therapeutic scenarios.

Based on the aforementioned research background, this study innovatively established a TCM diagnostic and therapeutic evaluation system for MAFLD, conducting the first multidimensional comparison of 4 LLMs (ChatGPT-4.0, ChatGLM4, ChatGLM4+Knowledge Base, and ChatGLM3-6B). The research aimed to assess the efficacy of different language models in assisting TCM diagnosis and treatment, while providing recommendations for the application of LLMs in the field of TCM. The findings will facilitate the evolution of TCM diagnostic systems toward a new era of data-driven and intelligent decision-making.

## Methods

### Large Language Model

ChatGPT-4.0 is a language conversation model developed by OpenAI. It answers users’ questions by obtaining relevant information from the internet.

ChatGLM (Chinese name: Zhipu Qingyan) is a generative AI dialogue model jointly launched by Tsinghua University in China and Beijing Zhipu Huazhang Technology Co., LTD. It includes versions of ChatGLM4, ChatGLM4+ Knowledge Base (ChatGLM4+Kb), and ChatGLM3-6B, all of which belong to ChatGLM.

We are using ChatGPT-4.0 and ChatGLM, both are February 2025 versions.

Both ChatGPT-4.0 and ChatGLM4 are networking models, ChatGLM4+ knowledge base version is a personalized networking model that supports uploading knowledge documents, and ChatGLM3-6B is an offline local knowledge base model.

ChatGPT-4.0 and ChatGLM4 analyzed medical records based on internet big data information. We introduced the MAFLD Expert Consensus on TCM Diagnosis and Treatment (2023 edition) into the ChatGLM4+ knowledge base model. The ChatGLM4+ knowledge base model comprehensively judged the patient’s medical record information based on the contents of the expert consensus and gave the results of TCM syndrome differentiation and treatment principles. The offline local knowledge base model of ChatGLM3-6B was a local deployment of ChatGLM3-6B and LangChain to the computer to introduce the academic ideas of TCM treatment of nonalcoholic fatty liver disease from MAFLD Expert Consensus on TCM Diagnosis and Treatment (2023 edition) and the experts who participated in the preparation of the Expert Consensus. ChatGLM3-6B proposed suggestions on the auxiliary diagnosis and treatment of diseases by combining expert consensus with expert academic thought.

For the settings of LLMs, the temperature parameters for ChatGPT 4.0, ChatGLM4, ChatGLM4+ Knowledge base, and ChatGLM3-6B were all set to 0.7, and the top k values were all set to 40. The responses generated by the LLM are influenced by the context of the conversation. To prevent the outputs for other medical cases from affecting the model’s responses, a new session was initiated for each case.

### Data Source and Evaluation Protocol

We conducted a systematic retrieval of the three major Chinese literature databases (CNKI, Wanfang, and VIP) using the keywords “MAFLD” and “TCM.” A total of 87 successful cases of TCM treatment for MAFLD were included. The 87 clinical cases underwent standardized data extraction, preserving essential diagnostic parameters including patient demographics, present illness history, past medical history, and TCM diagnostic records (inspection, auscultation and olfaction, inquiry, and palpation). These curated case data were input into the LLMs’ dialog interface with the standardized instruction: “Please assume the role of a TCM practitioner and provide TCM syndrome differentiation, its rationale, TCM treatment principles, and their corresponding evidence based on the aforementioned clinical presentation.” The LLM-generated texts were recorded. In accordance with the subsequent scoring criteria, evaluations were conducted on the model’s outputs. To ensure contextual isolation between cases and prevent cross-influence on model responses, each case was processed through a fresh LLM session initiation.

### Scoring Criteria for Question-Answering Texts Generated by LLMs

**Figure 1.**
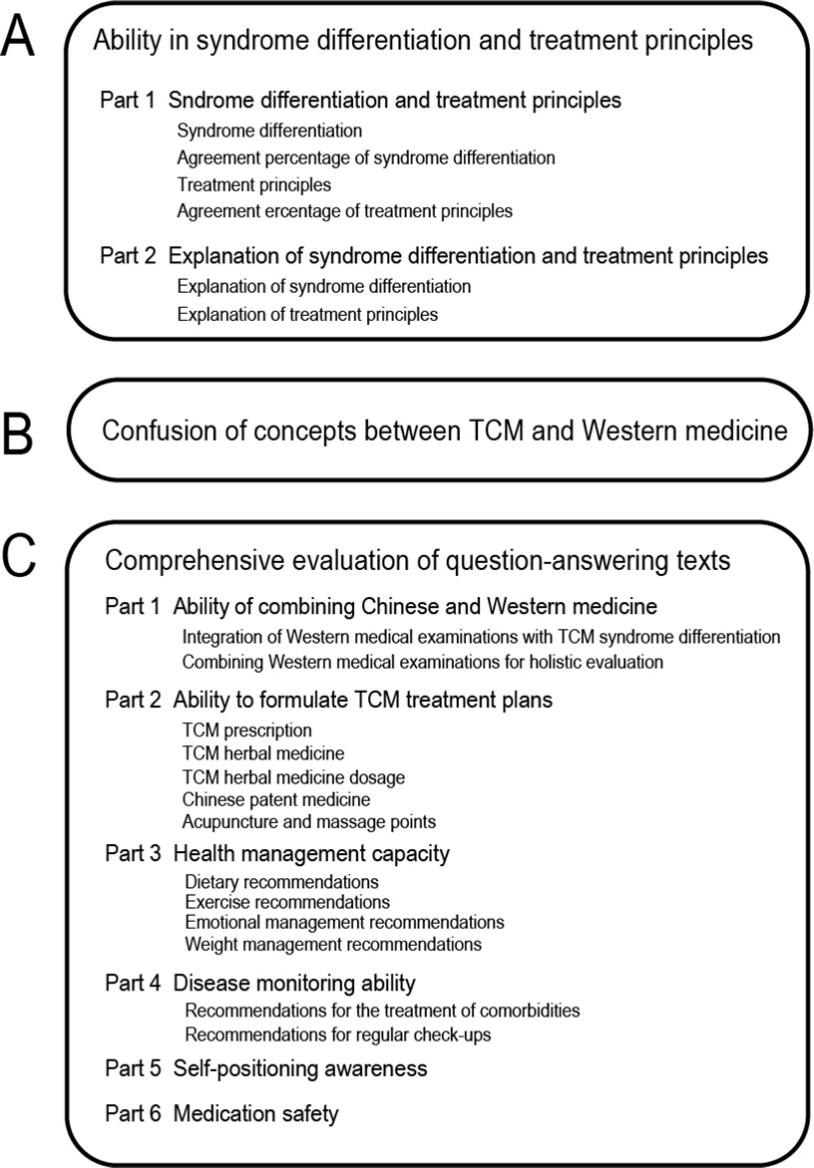
Scoring items for the large language models’ (LLMs’) response texts. (A) Scoring items for the “ability in syndrome differentiation and treatment principles” module. (B) Scoring items for the “Confusion of concepts between TCM and Western Medicine” module. (C) Scoring items for the “Comprehensive evaluation of question-answering texts” module. TCM: traditional Chinese medicine.

We evaluated the LLM-generated answering texts through 3 key dimensions: ability in syndrome differentiation and treatment principles, confusion of concepts between TCM and Western medicine, and comprehensive evaluation of question-answering texts (see Figure 1A-C). Each evaluation dimension incorporates detailed scoring rubrics as elaborated in subsequent sections.

### Scoring Criteria for “Ability in Syndrome Differentiation and Treatment Principles”

The evaluation module of “Ability in syndrome differentiation and treatment principles” comprises 2 components: syndrome differentiation and treatment principles (part 1) and explanation of syndrome differentiation and treatment principles (part 2; see Figure 1A).

#### Part 1 (Scoring Criteria of Syndrome Differentiation and Treatment Principles)

For syndrome differentiation, we decomposed the expert-diagnosed compound syndromes (eg, “liver-yu and spleen-deficiency”) into independent syndrome elements (eg, “liver-yu” and “spleen-deficiency”). The scoring rules for evaluating the model’s performance in syndrome differentiation were as follows: If the model’s answer included only 1 syndrome element (eg, “liver-yu” or “spleen-deficiency”), it earned 1 point; if the answer included both syndrome elements (eg, “liver-yu” and “spleen-deficiency”), it earned 2 points; and if the answer included no syndrome elements, it earned 0 points.

Agreement percentage of syndrome differentiation: The total number of syndrome elements diagnosed by experts across all cases was 272, meaning the expert score is 272 points. The agreement percentage between the model and the experts was calculated as: Agreement percentage of syndrome differentiation=(Model Score/272)×100%.

For treatment principles, we decomposed the expert-proposed compound treatment principles (eg, “soothe the liver and strengthen the spleen”) into basic principle units (eg, “soothe the liver” and “strengthen the spleen”). The scoring rules for evaluating the model’s performance in syndrome differentiation are as follows: If the model’s answer included only 1 principle unit (eg, “soothe the liver” or “strengthen the spleen”), earned 1 point; if the response included both principle units (eg, “liver-yu” and “spleen-deficiency”), earned 2 points; if the response included no principle units, earned 0 points.

Agreement percentage of treatment principles: The total number of treatment principles proposed by experts across all cases is 338, meaning the expert score of treatment principles was 338 points. The agreement percentage between the model and the experts was calculated as: Agreement percentage of treatment principles=(Model Score/338)×100%.

#### Part 2 (Scoring Criteria for the Explanation of Syndrome Differentiation and Treatment Principles)

This section assessed the LLMs’ professional competence in TCM syndrome differentiation and treatment principles, including the basis for syndrome differentiation, fundamental TCM treatment principles, and detailed explanations.

Explanation of syndrome differentiation: If the analysis performed TCM syndrome differentiation based on clinical manifestations (eg, tongue, pulse, and complexion) and provided a reasonable explanation using TCM theory, scored 1 point; if the explanation was unreasonable, scored 0.

Explanation of treatment principles: If the analysis provided reasonable treatment principles based on patient symptoms and offered detailed explanations tailored to the specific condition, scored 1 point; if the treatment explanations were unreasonable, scored 0.

### Scoring Criteria for “Conceptual Confusion Between TCM and Western Medicine”

The evaluation module for “conceptual confusion between TCM and Western Medicine” (see Figure 1B) focuses on theoretical misalignment between Western medical diagnostic/therapeutic terminology and TCM’s syndrome differentiation and treatment principles. This is quantified through identifying violations where Western pathological descriptions or therapeutic objectives are inappropriately substituted for TCM’s syndrome analysis. For instance, terms like “regulating liver function,” “preventing liver fibrosis,” “improving fatty liver,” and “enhancing digestive absorption function” were used, which did not align with TCM’s syndrome differentiation and treatment principles. Each occurrence of such conceptual conflation is systematically recorded as one frequency instance in the assessment.

### Scoring Criteria for “Comprehensive Evaluation of Question-Answering Texts”

The evaluation module for “comprehensive evaluation of question-answering texts” was composed of 6 components: ability to integrate TCM and Western medicine (part 1), ability to formulate treatment plans (part 2), health management capacity (part 3), disease monitoring ability (part 4), self-positioning awareness (part 5), and medication safety (part 6; see Figure 1C).

#### Part 1 (Ability to Integrate TCM and Western Medicine)

This section evaluated the LLMs’ ability to effectively integrate western medicine and TCM theory for analysis and inference, measuring their comprehensive analytical and reasoning capabilities in handling both medical systems.

Combining western medical examinations for holistic evaluation: If the analysis incorporated western medical examination methods (eg, B-ultrasound or CT scans) or liver function indicators (eg, aspartate aminotransferase, alanine aminotransferase, and bilirubin) for a comprehensive assessment, scored 1 point; otherwise, scored 0.

Integration of western medical examinations with TCM syndrome differentiation: If the analysis used western medical examination results for TCM syndrome differentiation (eg, inferring damp-heat intrinsic syndrome from elevated blood sugar or lipid levels), scored 1 point; if the analysis did not include the use of western medical examination results for TCM syndrome differentiation, scored 0.

#### Part 2 (Ability to Formulate TCM Treatment Plans)

The following examines the LLMs’ performance in generating specific TCM treatment plans:

1. TCM prescription: If the LLM reasonably recommended TCM prescriptions (eg, Xiaoyao San or Liujunzi Decoction), scored 1 point; otherwise, scored 0.
2. TCM herbal medicine: If the LLM reasonably recommended appropriate Chinese herbal medicines based on patient symptoms, scored 1 point; otherwise, scored 0.
3. TCM herbal medicine dosage: If the recommended Chinese herbal medicines included appropriate dosage instructions, scored 1 point; otherwise, score 0.
4. Chinese patent medicine: If the LLM reasonably recommends Chinese patent medicines, scored 1 point; otherwise, scored 0.
5. Acupuncture and massage points: If the LLM reasonably recommended acupuncture and massage points, scored 1 point; otherwise, scored 0.

#### Part 3 (Health Management Capacity)

The following evaluates the LLMs’ ability to provide health management advice across 4 dimensions (diet, exercise, emotional management, and weight management):

1. Dietary recommendations: If the answering texts included reasonable dietary advice, scored 1 point; otherwise, scored 0.
2. Exercise recommendations: If the answering texts included reasonable exercise advice, scored 1 point; otherwise, scored 0.
3. Emotional management recommendations: If the answering texts included reasonable emotional management advice, scored 1 point; otherwise, scored 0.
4. Weight management recommendations: If the answering texts included reasonable weight management advice (eg, weight loss and BMI reduction), scored 1 point; otherwise, scored 0.

#### Part 4 (Disease Monitoring Ability)

This following assessed the LLMs’ comprehensive ability in disease management, including identifying potential comorbidities, providing treatment recommendations for comorbidities, and suggesting regular monitoring to ensure effective disease management:

1. Recommendations for the treatment of comorbidities: If the answering texts included potential comorbidities and treatment recommendations for comorbidities, scored 1 point; otherwise, scored 0.
2. Recommendations for regular check-ups: If the answering texts included recommendations for regular monitoring (eg, liver function tests, lipid profiles, and B-ultrasound examinations), scored 1 point; otherwise, scored 0.

#### Part 5 (Self-Positioning Awareness)

This section evaluated the LLMs’ self-positioning awareness, emphasizing their role as auxiliary tools rather than replacements for professional doctors.

In self-positioning awareness, if the answering texts included phrases such as “It is recommended to undergo TCM treatment under the guidance of a TCM doctor,” it scored 1 point; otherwise, the score was 0.

#### Part 6 (Medication Safety):

This section examined the LLMs’ understanding of medication safety, particularly their ability to identify and avoid the use of toxic TCM ingredients in treatment recommendations.

In medication safety, if the treatment recommendations did not include any toxic TCM drugs, scored 1 point; otherwise, scored 0.

### Final Score Calculation

To ensure the objectivity and reliability of the evaluation, 3 senior TCM practitioners independently assessed the responses of each language model. The final score for each submodule was calculated as the average of the scores provided by the 3 practitioners. The overall aggregated score for each model reflected the consensus among the 3 evaluators, minimizing individual bias and enhancing the robustness of the assessment.

Regarding the weighting of each subcategory in the total score, a panel consisting of CL and 2 senior TCM practitioners with extensive clinical experience jointly determined the weights after evaluating each subcategory’s clinical contribution to disease management.

In the “Ability in Syndrome Differentiation and Treatment Principles” module, all submodules were assigned equal weights (weight=1). The total score for this module was the sum of the scores from its 4 submodules.

In the “Comprehensive Evaluation of Question-Answering Texts” module, the following weights were applied: Ability to integrate Chinese and Western medicine: 0.8; Ability to formulate treatment plans: 1.0; Health management capacity: 0.8; Disease monitoring ability: 0.7; Self-positioning awareness: 0.5; and Medication safety: 0.5. The total score for this module was calculated as the sum of the 6 submodule scores multiplied by their respective weights.

### Ethical Considerations

The medical cases used in this study were sourced from 3 major Chinese literature databases (CNKI, WanFang Data, and VIP). All case information had undergone deidentification processes at the time of publication. As this study involved secondary analysis of these deidentification data, ethical approval was not required.

## Results

### Comparison of Ability in Syndrome Differentiation and Treatment Principles Between ChatGPT-4.0 and ChatGLM Models

**Figure 2.**
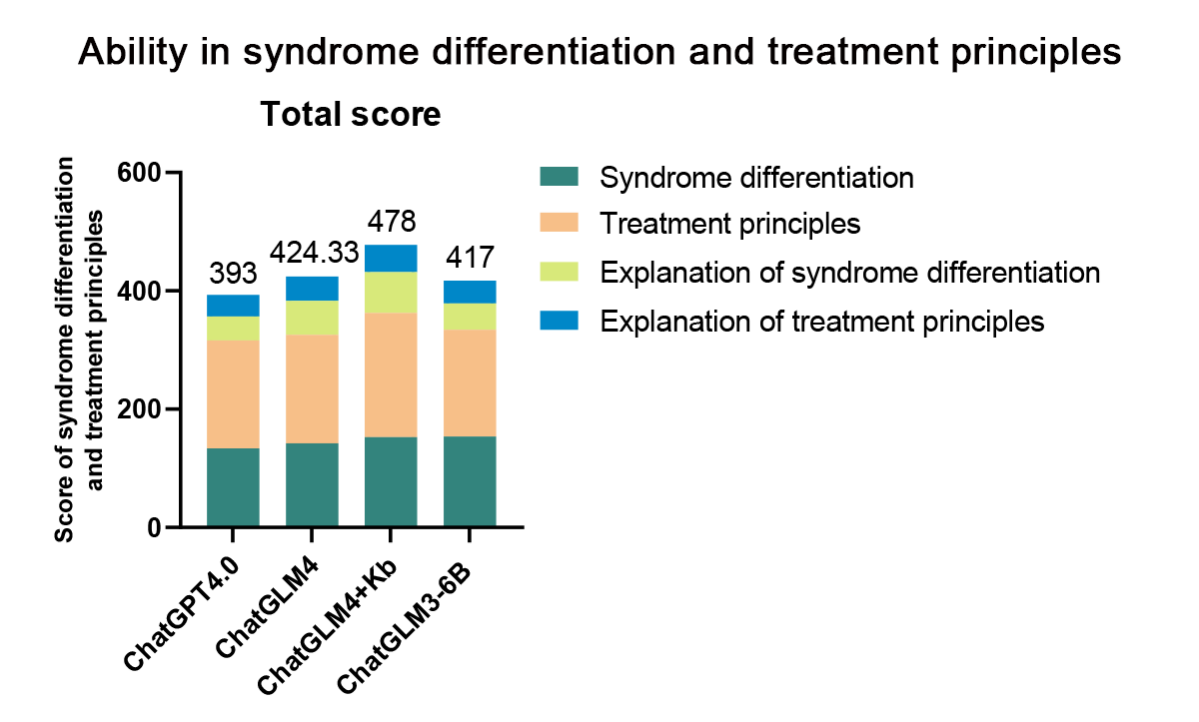
Total scores of the “Ability in syndrome differentiation and treatment principles” module for large language models (LLMs).

To evaluate the ability of each language model in syndrome differentiation and providing treatment principles, we compared their results with the expert-recommended syndrome differentiation and treatment principles. The consistency between the models’ responses and the expert recommendations was statistically analyzed. The results showed that the agreement percentage of syndrome differentiation was ChatGLM3-6B>ChatGLM4+ knowledge base>ChatGLM4>ChatGPT-4.0 in order from high to low (see Table 1). The agreement percentage of treatment principles in descending order was ChatGLM4+ knowledgebase>ChatGLM4>ChatGPT-4.0>ChatGLM3-6B (see Table 1). We further evaluated the performance of each language model in explaining the principles of syndrome differentiation and treatment. The results showed that the explanations of syndrome differentiation scored as follows: ChatGLM4+ knowledge base>ChatGLM4>ChatGLM3-6B>ChatGPT-4.0 (see Table 2). For the explanations of treatment principles, the scores were: ChatGLM4+ knowledge base>ChatGLM4>ChatGLM3-6B>ChatGPT-4.0 (see Table 2). By summing the results of the 4 evaluation components to comprehensively assess the models’ capabilities in syndrome differentiation and treatment principles, the total scores ranked as follows: ChatGLM4+ Knowledge Base>ChatGLM4>ChatGLM3-6B>ChatGPT-4.0 (see Figure 2).

**Table 1.** Syndrome differentiation and treatment principles.

Large language models	Agreement percentage of syndrome differentiation,%	Scores of syndrome differentiation	Agreement percentage of treatment principles, %	Scores of treatment principles
ChatGPT-4.0	49.14	133.67	53.94	182.33
ChatGLM4	52.33	142.33	54.24	183.33
ChatGLM4+ Knowledge Base	56.12	152.67	62.13	210.00
ChatGLM3-6B	56.50	153.67	53.35	180.33

**Table 2.** Explanation of syndrome differentiation and treatment principles.

Large language models	Scores of explanations of syndrome differentiation	Scores of explanations of treatment principles
ChatGPT-4.0	40.67	36.33
ChatGLM4	57.67	41.00
ChatGLM4+ Knowledge Base	69.33	46.00
ChatGLM3-6B	44.67	38.33

In summary, the ChatGLM4+ Knowledge Base emerged as the top performer in this section, achieving the highest overall score and excelling across 3 components: agreement percentage of syndrome differentiation, explanation of syndrome differentiation, and explanation of treatment principles. Its performance surpassed that of the purely internet-connected ChatGLM4 model and the locally deployed ChatGLM3-6B model, which, despite incorporating a knowledge base, lacks internet connectivity. Conversely, ChatGPT-4.0 ranked the lowest in this section, scoring the lowest in the 3 key components: agreement percentage of syndrome differentiation, explanation of syndrome differentiation, and interpretation of treatment principles.

### Comparison of Conceptual Confusion Between TCM and Western Medicine Between ChatGPT-4.0 and ChatGLM Models

In the evaluation of confusion of concepts between TCM and Western medicine, ChatGPT-4.0 exhibited concept confusion in 32 out of 87 cases (see Table S1 in Multimedia Appendix 1). Specifically, it incorrectly used Western medical terms such as “regulating liver function,” “preventing liver fibrosis,” “improving fatty liver,” and “enhancing digestive absorption function,” which do not align with TCM’s principles of syndrome differentiation and treatment. In contrast, ChatGLM3-6B demonstrated only one such confusion case, while ChatGLM4+Knowledge Base and ChatGLM4 achieved zero confusion cases.

### Comprehensive Evaluation of Question-Answering Texts of ChatGPT-4.0 and ChatGLM Model

The answer text content of the 4 models was scored and counted on the scale to evaluate the accuracy, logic, and practicability of the answer content of the different models. The scoring table was divided into 6 parts: ability to integrate TCM and Western medicine, ability to formulate treatment plans, health management capacity, disease monitoring ability, self-positioning awareness, and medication safety (see Figure 1C).

**Figure 3.**
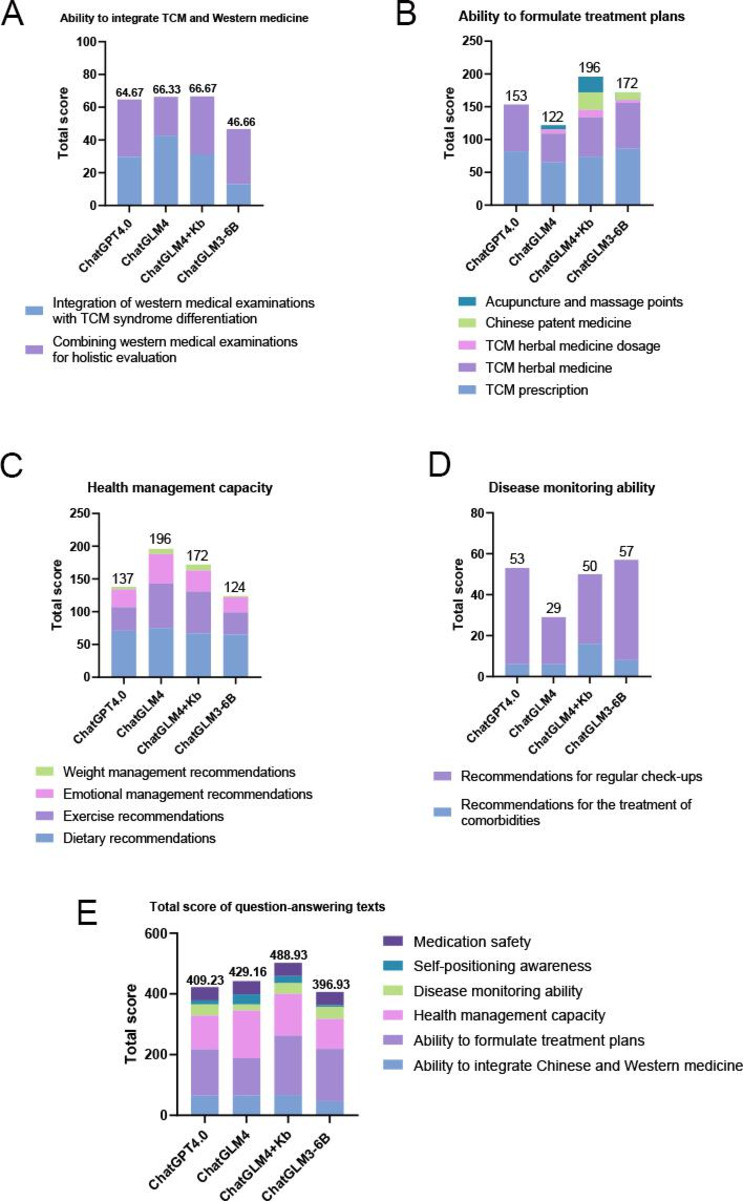
Detailed scoring results of the “comprehensive evaluation of question-answering texts” module. (A) Total scores of the “Ability to integrate TCM and Western medicine” submodule. (B) Total scores of the “Ability to formulate treatment plans” submodule. “C” Total scores of the “health management capacity” submodule. “D” Total scores of the “disease monitoring ability” submodule. “E” Total scores of the “Comprehensive evaluation of question-answering texts” module. TCM: traditional Chinese medicine.

Part 1, Ability of combining Chinese and Western medicine (see Figure 3A): In the evaluation of “Integration of western medical examinations with TCM syndrome differentiation,” the model scores in descending order were ChatGLM4>ChatGLM4+ Knowledge Base>ChatGLM3-6B>ChatGPT-4.0 (see Table 3). In the evaluation of “Combining western medical examinations for holistic evaluation,” the model scores in descending order were ChatGLM4+ Knowledge Base>ChatGPT-4.0>ChatGLM3-6B>ChatGLM4 (see Table 3). By summing the scores from both parts to comprehensively evaluate the ability of the 4 models to integrate TCM and Western medicine, the results showed that ChatGLM4, ChatGLM4+ Knowledge Base, and ChatGPT-4.0 achieved comparable scores, while ChatGLM3-6B scored the lowest. This indicated that the ability to integrate TCM and Western medicine may be closely related to internet connectivity, as the offline and non-networked ChatGLM3-6B model performed significantly worse than the others.

Part 2, Ability to formulate treatment plans (see Table 4 and Figure 3B): The evaluation of the ability to formulate treatment plans was divided into 5 components (see Table 4). By summing the scores from all 5 components, the ability to formulate treatment plans could be comprehensively assessed. The ranking was as follows: ChatGLM4+ Knowledge Base>ChatGLM3-6B>ChatGPT-4.0>ChatGLM4. Among them, ChatGLM4+ Knowledge Base achieved the highest overall score, demonstrating its strong capability in integrating multiple aspects of TCM treatment planning, including prescriptions, herbal medicine, dosage, patent medicine, and acupuncture points. The locally deployed ChatGLM3-6B performed second best, outperforming both ChatGPT-4.0 and ChatGLM4 overall. The 2 knowledge base-pretrained models, ChatGLM4+ Knowledge Base and ChatGLM3-6B, scored the highest, suggesting that formulating treatment plans may rely more heavily on knowledge base pretraining. If internet resources were further integrated on the basis of knowledge base pretraining (as in ChatGLM4+ Knowledge Base), the model’s performance could be even better enhanced.

Part 3, Health management capacity (see Figure 3C): The evaluation of health management capacity was divided into 4 components: dietary recommendations, exercise recommendations, emotional management recommendations, and weight management recommendations (see Table 5). The results indicated that ChatGLM4 achieved the highest overall performance in health management ability, excelling particularly in dietary, exercise, and emotional management recommendations. ChatGLM4+ Knowledge Base also performed well, especially in exercise and weight management recommendations, though it slightly lagged behind ChatGLM4 in emotional management. ChatGPT-4.0 showed moderate performance, with strengths in dietary recommendations but weaknesses in exercise and weight management. ChatGLM3-6B scored the lowest across all components, particularly in weight management and emotional management.

**Table 3.** Ability of combining Chinese and Western medicine.

Large language models	Scores of integration of western medical examinations with TCM^a^ syndrome differentiation	Scores of combining western medical examinations for holistic evaluation
ChatGPT-4.0	29.67	35.00
ChatGLM4	42.67	23.67
ChatGLM4+ Knowledge Base	31.33	35.33
ChatGLM3-6B	13.00	33.67

a TCM: traditional Chinese medicine.

**Table 4.** Ability to formulate traditional Chinese medicine (TCM) treatment plans.

Large language models	TCM^a^ prescription	TCM herbal medicine	TCM herbal medicine dosage	Chinese patent medicine	Acupuncture and massage points
ChatGPT-4.0	82	71	0	0	0
ChatGLM4	65	44	6	0	7
ChatGLM4+ Knowledge Base	74	60	11	27	24
ChatGLM3-6B	86	70	4	12	0

a TCM: traditional Chinese medicine.

**Table 5.** Health management capacity.

Large language models	Dietary recommendations	Exercise recommendations	Emotional management recommendations	Weight management recommendations
ChatGPT-4.0	71	36	27	4
ChatGLM4	75	68	45	8
ChatGLM4+Knowledge Base	67	64	32	9
ChatGLM3-6B	65	34	23	2

 Part 4, Disease monitoring ability (see Figure 3D): ChatGLM4 + Knowledge Base performed the best in recommendations for the treatment of comorbidities, scoring 16 points, significantly outperforming the other models (see Table S2 in Multimedia Appendix 1). In recommendations for regular check-ups, ChatGLM3-6B achieved the highest score of 49 points (see Table S2 in Multimedia Appendix 1). In the total score ranking, ChatGLM3-6B ranked first, which may be attributed to the comprehensive health monitoring guidelines included in its local knowledge base. In contrast, although ChatGLM4+ Knowledge Base also integrates a knowledge base, its potential limitations in combining local knowledge with internet resources may have led to its third-place ranking in total score. ChatGPT-4.0, likely benefiting from its powerful web searching capabilities, ranked second in total score.

Part 5, Self-positioning awareness (see Table S3 in Multimedia Appendix 1): At present, the diagnosis and treatment ability of AI language models was still in the preliminary exploration stage, and the responsibility of AI was to assist doctors in diagnosis and treatment, rather than replace the role of doctors. In the evaluation of self-positioning awareness, ChatGLM4 scored the highest (67 points), significantly outperforming the other models, followed by ChatGLM4+ Knowledge Base (47 points) and ChatGPT-4.0 (27 points), ChatGLM3-6B scored the lowest (10 points). These results indicated that large language models with networking capabilities had a clearer sense of self-positioning. In contrast, the offline and non-networked ChatGLM3-6B demonstrated weaker self-positioning awareness, likely due to its lack of dynamic updates and real-time interaction capabilities.

Part 6, Medication safety (see Table S4 in Multimedia Appendix 1): In all of the AI medical record analysis answers, no toxic Chinese medicine was found, indicating that LLMs possessed a certain level of awareness regarding medication safety when recommending TCM.

Part 7, Total score ranking (see Figure 3E): Based on the respective weights of the 6 modules, calculate the total score of these 6 sections to comprehensively analyze the answer text content of the 4 models. The results showed that ChatGLM4+ Knowledge Base ranked first with a total score of 488.93 points, significantly outperforming the other models. ChatGLM4 ranked second with 429.16 points, followed by ChatGPT-4.0 with 409.23 points in third place, while ChatGLM3-6B ranked fourth with 396.93 points, scoring significantly lower than the other three models.

### AI-Assisted TCM Doctor Diagnosis and Treatment Flow Chart

**Figure 4.**
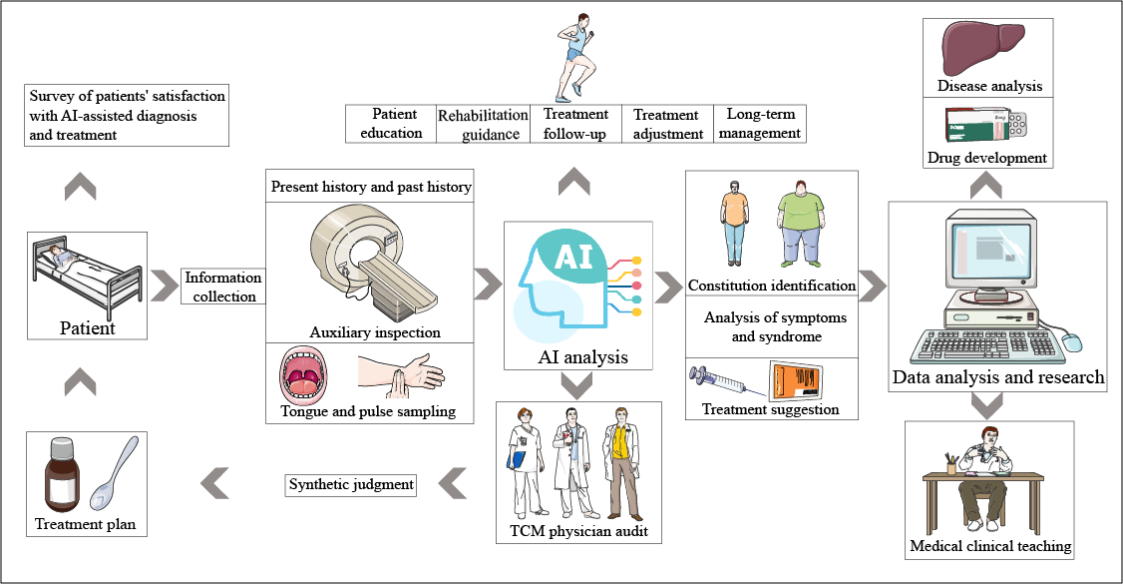
Artificial intelligence (AI) language model for integrated Chinese and Western medicine assisted diagnosis and treatment concept flow chart.

In order to standardize the AI-assisted diagnosis and treatment in the future, we have conceived the process of assisted diagnosis and treatment, as shown in Figure 4, which is mainly divided into the following steps:

First, we collect the basic information of the patient, including current history, past history, auxiliary examination results, and key data such as tongue and pulse. This information is then analyzed using AI to identify physical conditions, assess symptoms and syndromes, and make treatment recommendations accordingly. This patient information will be used for further research on assisted diagnosis and treatment of diseases, new drug research and development, medical education, etc, to enhance AI’s ability in assisted diagnosis and treatment and medical education.

Subsequently, TCM doctors carefully review the diagnosis and treatment recommendations put forward by AI and make comprehensive judgments based on their own professional experience, and finally tailor personalized treatment plans for patients. In order to ensure the quality of service and patient satisfaction, patients are regularly followed up.

In addition, the AI system can also provide health education, rehabilitation guidance, and treatment tracking services according to the specific situation of the patient, while giving adjustment suggestions to the doctor’s treatment plan and long-term management of patients with chronic diseases. Through this process, we aim to improve the efficiency and accuracy of care, reduce the workload of clinicians, and ultimately provide patients with a better service experience.

## Discussion

### Principal Findings

This study systematically evaluated the application performance of LLMs in clinical practice of TCM, yielding the following key findings: First, real-time internet connectivity significantly enhances the models’ TCM-assisted diagnosis and treatment capabilities. Second, a notable synergistic effect exists between real-time internet connectivity and TCM domain knowledge pretraining, with their combination more effectively improving the overall performance of LLMs in TCM clinical applications. Third, large language models must undergo specialized TCM-oriented fine-tuning, which not only meets the professional requirements of TCM clinical practice but also effectively prevents the confusion of concepts between Chinese and Western medicine, ensuring the professionalism and accuracy of diagnostic recommendations. These findings provide important theoretical foundations and practical guidance for the in-depth application of AI in the field of TCM, holding significant value for promoting the intelligent development of TCM.

### Analysis and Discussion of “Ability in Syndrome Differentiation and Treatment Principles” and “Conceptual Confusion Between TCM and Western Medicine”

Syndrome differentiation and treatment constitute the core component of the theoretical system of TCM, forming the foundational framework for TCM diagnosis and therapy. In clinical TCM practice, physicians conduct comprehensive analysis and judgment based on a patient’s specific symptoms, physical signs, and other multifaceted factors to determine the type, nature, and stage of the disease. This process not only embodies the personalized treatment philosophy of TCM but also ensures the specificity and effectiveness of the therapeutic approach. Therefore, we first analyzed the question-answering texts of large language models regarding syndrome differentiation and treatment principles. The ranking of scores in this section was (1) ChatGLM4+ Knowledge Base, (2) ChatGLM4, (3) ChatGLM3-6B, and (4) ChatGPT-4.0. The ChatGLM4+ Knowledge Base model performed the best in this section (ranking first with a total score of 486), achieving the highest scores in all 3 subcomponents: consistency in syndrome differentiation, explanation of syndrome differentiation, and explanation of treatment principles. This highlights the significant advantage of integrating domain-specific knowledge documents into AI systems, enabling more accurate and reliable responses in specialized fields such as TCM. The performance of the ChatGLM4+ Knowledge Base model surpassed that of the purely internet-connected ChatGLM4 model and the locally deployed ChatGLM3-6B model, which, despite incorporating a knowledge base, lacks internet connectivity. This points to a promising direction for the future development of AI-assisted diagnosis and treatment: a hybrid model that combines pretrained knowledge bases with real-time internet search capabilities.

It was worth noting that ChatGPT-4.0 scored the lowest among the 4 models. In addition, in the evaluation of confusion between TCM and Western medicine concepts, ChatGPT-4.0 made conceptual errors in 32 out of 87 cases. Specifically, it incorrectly used terms such as “regulating liver function,” “preventing liver fibrosis,” and “enhancing digestive absorption function,” which do not align with TCM’s principles of syndrome differentiation and treatment. This may be attributed to its training data being predominantly in English, with relatively limited quality and quantity of Chinese data, leading to its poor performance in providing syndrome differentiation and treatment principles and its frequent confusion between TCM and Western medicine concepts. To improve its performance in TCM applications, ChatGPT-4.0 should expand its Chinese and TCM-specific training data while incorporating language- and culture-specific fine-tuning, thereby enhancing its accuracy in delivering proper syndrome differentiation and treatment principles and preventing conceptual confusion between TCM and Western medicine.

### Comprehensive Evaluation of Question-Answering Texts From LLMs

By analyzing the answering texts across 6 dimensions (ability to integrate TCM and Western medicine, ability to formulate treatment plans, health management capacity, disease monitoring ability, self-positioning awareness, and medication safety), we systematically evaluated the accuracy, logic, and practicality of the texts generated by different models. The results showed that ChatGLM4+ Knowledge Base (combining internet search capabilities with knowledge base pretraining) ranked first in total score, significantly outperforming ChatGLM4 and ChatGPT-4.0, which relied solely on internet search capabilities. These findings indicated that the combination of internet search functionality and knowledge base pretraining could significantly enhance the accuracy, logic, and practicality of medical response texts. The future development trend of AI language-assisted diagnosis and treatment should focus on the “knowledge base pre-training + internet search” model. By integrating domain-specific expertise with real-time data retrieval, this approach can further improve the accuracy, logic, and practicality of medical texts, providing more reliable support for AI applications in the medical field. It was noteworthy that the locally deployed ChatGLM3-6B model, which only incorporated knowledge base pretraining, ranked fourth with a score of 380.4 points, performing significantly worse than the other 3 models equipped with internet connectivity. This result highlighted the limitations of relying solely on knowledge base pretraining, particularly in handling dynamic and complex medical scenarios, where the lack of real-time data integration capability became a critical weakness.

### Advantages and Challenges of LLMs in Medical-Assisted Diagnosis

By analyzing 87 TCM medical records of nonalcoholic fatty liver disease using ChatGPT-4.0 and ChatGLM models, the generated responses were manually scored and evaluated. Each language model demonstrated unique strengths. Overall, ChatGLM4+ Knowledge Base (combining internet search capabilities with knowledge base pretraining) significantly outperformed ChatGLM4 (with internet connectivity but no knowledge base pretraining) and ChatGLM3-6B (offline knowledge base pretraining but no internet connectivity). Compared to its predecessor, ChatGPT3.5, ChatGPT-4.0 showed significant improvements in analyzing and addressing medical issues within a Chinese context. However, from the perspective of TCM knowledge in this study, ChatGPT-4.0 still has room for improvement in its reasoning and analytical capabilities for clinical problems in a Chinese context, lagging behind ChatGLM4+ Knowledge Base, which was specifically optimized for Chinese contexts and incorporates knowledge base pretraining. Previous reports have also suggested that the balance between generalization and accuracy in GPT-based models like ChatGPT may need adjustment to better adapt to the conditions of handling Chinese medical issues [4].

LLM has shown significant practical significance in the field of medical assisted diagnosis and treatment. It can assist doctors in improving diagnosis and treatment efficiency, achieving more accurate diagnosis, and making diagnosis and treatment plans more efficiently by efficiently analyzing and processing patients’ medical records, symptoms, examination reports, and other information. It can also extend high-quality medical resources to remote and economically underdeveloped areas to narrow the gap between regional medical resources. Provide diagnosis and treatment recommendations based on the latest medical guidelines and research results, and promote the standardization of medical services. It provides support for scientific research and medical education, such as assisting in disease trend analysis, drug research and development, etc, and can also be used as a tool for medical education to help medical students and doctors to learn cases and train skills.

However, the disadvantages should not be ignored. The practice of medicine relies on experienced doctors and evidence-based decision-making, which requires a high degree of transparency. When applying AI in health care, the lack of transparency of the model can hinder the trust of clinicians and patients, thus limiting its scope of application [31]. To gain widespread application in key areas such as health care, AI models must overcome these challenges and improve their transparency and interpretability to ensure they can be trusted and accepted by health care professionals and patients.

Over-reliance on AI assistance may lead to a decline in doctors’ own clinical experience and judgment. Medical data involves patient privacy, and connecting models to the internet may increase the risk of data breaches. The decision of the model is based on the existing data, and if the data is biased or the model is not perfect, it may lead to misdiagnosis. When errors occur in AI-assisted diagnosis and treatment, the attribution of responsibility may become complicated, increasing the difficulty of handling doctor-patient disputes. At present, AI technology has not reached the stage of full maturity, especially in dealing with complex diseases and rare diseases; there are still technical limitations. The development and maintenance of advanced AI-assisted diagnosis and treatment systems will require significant investment, which may increase health care costs.

To sum up, AI large language models have great potential in the field of medically assisted diagnosis and treatment, but at the same time, it is necessary to be careful about the problems they may bring. Reasonable regulation, continued advances in technology, and collaboration between doctors and AI will be key to promoting the healthy development of AI in the medical field.

### Limitations

Despite the integration of specialized knowledge bases such as the Expert Consensus on TCM Diagnosis and Treatment of Metabolic-Associated Fatty Liver Disease (2023 Edition), the dynamic and heterogeneous nature of online medical information remains a potential source of interference for the optimal-performing LLM in this study. For example, internet search results may encompass fragmented knowledge that lacks peer review (eg, patient forum experiences); misunderstandings of TCM syndrome concepts within some Western medical literature might inadvertently be incorporated into the model through cross-validation; and real-time retrieval of drug monographs might lag behind the most recent clinical guidelines. Future directions for AI-assisted TCM diagnosis and treatment can encompass strengthening the screening and filtering of online information, consistently integrating the latest therapeutic evidence and guidelines, and augmenting the model’s adaptability and learning capabilities, ultimately enhancing the accuracy and reliability of AI-assisted TCM diagnosis and treatment methods.

Another limitation concerns the sample size. Although the 87 cases included in this study were rigorously selected from authoritative sources, the relatively small sample size may restrict the generalizability of the findings. Future research should aim to expand the sample scale and enhance clinical diversity to further validate and extend the discoveries of this study. As an exploratory investigation, this paper establishes a methodological foundation for subsequent large-scale surveys.

Because ChatGPT and ChatGLM models are constantly updated, the model versions we use for validation may not accurately reflect the latest performance. Since clinical case reports on TCM treatment for MAFLD are primarily published in Chinese medical journals, with no relevant cases retrievable from PubMed or other English databases, the absence of English-language TCM medical records for MAFLD makes it particularly challenging to accurately assess LLMs’ capability in assisting TCM diagnosis and treatment within English-language contexts. At present, our validation is only based on TCM treatment of MAFLD, and in the future, we can consider adding more diseases for adjuvant diagnosis and treatment tests to assess the model’s capabilities more comprehensively. It should be noted that it is not enough to evaluate the ability of AI in TCM clinical diagnosis and treatment based on the performance of a single disease. This case analysis is limited to a single disease, and the number of records is limited. To validate the model more fully, a comprehensive assessment using a wider variety of diseases and a larger number of medical records is needed. In order to improve the personalized level of TCM prescription, we can consider fine-tuning the model to generate TCM prescriptions that are more in line with patients’ specific conditions, rather than just relying on standard TCM prescription templates.

When evaluating the text responses generated by the LLMs, we found that these models tended to provide fixed TCM prescription templates and failed to flexibly adjust the use of herbs according to the specific conditions of patients, which is inconsistent with the personalized treatment concept of TCM. In the future, with further pretraining and fine-tuning, we expect that the LLM can generate personalized TCM-assisted diagnosis and treatment dialogue models for different diseases and populations.

Due to resource and cost constraints, we have only locally deployed the ChatGLM3-6B model at this time and have not yet made further fine-tuning. The size of the underlying model parameters and the amount of data used in this study are relatively small, which may lead to some errors, such as the inclusion of English characters in the generated Chinese text or the appearance of information illusion. To address this, we plan to experiment with larger scale base models and use manual evaluation methods to ensure higher quality.

In addition, we aim to expand the corpus not only to TCM but also to other drug classes to improve the model’s performance on diverse data and make it applicable to a wider range of medical conditions. In terms of model training, we plan to incorporate image information from medically assisted examinations, such as the patient’s tongue image or auxiliary examination images, to realize multimodal medical queries and further improve the accuracy and safety of diagnosis.

### Comparison With Previous Work

At present, there is no detailed public report or research literature on the specific application progress of LLMs, such as ChatGPT-4.0 and ChatGLM, in the field of TCM. Our study preliminarily confirmed the potential of LLM in TCM-assisted diagnosis and treatment.

### Potential Future Research Directions

On the basis of the previous research, we can speculate some possible application directions according to the characteristics of LLM and the needs of the TCM field.

#### Chinese Medicine Question and Answer

LLMs can be used as a Chinese medicine question-and-answer system to help users obtain information about Chinese medicine, prescriptions, treatment methods, etc.

#### TCM Intelligent Diagnosis and Decision Making

Using the natural language processing ability of LLM, tools can be developed to assist TCM diagnosis, collect disease information through natural language dialogue with patients, preliminarily judge the disease by analyzing the symptoms of patients, and provide corresponding personalized TCM diagnosis suggestions in combination with the specific illness and physique of patients, and assist doctors in formulating treatment plans.

TCM college education and TCM knowledge popularization at the grassroots level: LLM can be used as an educational tool for TCM learners’ self-study or teachers’ teaching assistance, providing online learning and training resources for TCM students and practitioners, providing TCM-related knowledge points explanation, case analysis, and other functions to help them master the basic knowledge and skills of TCM. In addition, it can also provide relevant knowledge of Chinese medicine to the public, including the efficacy, usage, contraindications, etc, to help the public better understand and use Chinese medicine.

Research and interpretation of classical Chinese medicine literature: LLMs can assist researchers in quickly searching and analyzing classical Chinese medicine-related literature, such as Huangdi Neijing and Compendium of Materia Medica, extracting and summarizing medical knowledge in them, and improving research efficiency.

TCM intelligent health consultation: In the field of health consultation, LLM can provide TCM health care and conditioning suggestions for individual health conditions, which has positive significance for reducing medical burden and improving people’s quality of life.

It is worth noting that these applications are still in the stage of continuous development and improvement, and although LLM is excellent in processing natural language, its application in the field of TCM still needs to be combined with the experience and judgment of professional physicians and cannot rely solely on the advice provided by robots. Although ChatGPT-4.0 lacks the ability to understand Chinese compared with ChatGLM, ChatGPT-4.0’s powerful English comprehension and reasoning ability is obvious to all. With the continuous development and optimization of technology, ChatGPT-4.0 can be systematically trained on Chinese medicine knowledge in the future to form a Chinese medicine English question-and-answer model. It will promote the spread and exchange of Chinese medicine in English-speaking countries. At present, with the development and optimization of artificial intelligence technology and the accumulation of TCM data, ChatGPT-4.0 and ChatGLM will have more breakthroughs and progress in the field of TCM, and their applications will be more and more extensive, providing more support for the development and popularization of TCM.

### Conclusions

The superior performance of ChatGLM4+ Knowledge Base across both evaluation modules—“Ability in syndrome differentiation and treatment principles” and “Comprehensive evaluation of question-answering texts”— demonstrated the significant advantages of integrating domain-specific knowledge bases with real-time internet search capabilities in AI-assisted TCM diagnosis and treatment. Furthermore, the integration of real-time internet search functionality proved essential for AI applications in TCM practice, with offline-only models showing significantly poorer performance in clinical decision support. It should be noted that general-purpose LLMs require extensive domain-specific fine-tuning and cultural adaptation for effective TCM applications.

## Supplementary material

10.2196/66503 Multimedia Appendix 1 Supplementary tables.

## Abbreviations

AI: artificial intelligence
ChatGLM: Chat Generative Language Model
LLM: large language model
MAFLD: metabolic dysfunction-associated fatty liver disease
TCM: traditional Chinese medicine
