# Supplementary material for: Performance Assessment of ChatGPT-4.0 and ChatGLM Series in Traditional Chinese Medicine for Metabolic Associated Fatty Liver Disease: Comparative Study
Source: JMIR Form Res. 2025 Aug 25;9:e66503. doi: 10.2196/66503 (PMC12377871; doi:10.2196/66503)
Supplement: Multimedia Appendix 1 [file formative-v9-e66503-s001.docx]

**Table S1.** Confusion of concepts between traditional Chinese medicine (TCM) and Western medicine.

| Large language models | Confusion of concepts between TCM and Western medicine (frequency) |
| --- | --- |
| ChatGPT4.0 | 32 |
| ChatGLM4 | 0 |
| ChatGLM4+Knowledge Base | 0 |
| ChatGLM3-6B | 1 |

**Table S2.** Disease monitoring ability.

| Large language models | Recommendations for the treatment of comorbidities | Recommendations for regular check-ups |
| --- | --- | --- |
| ChatGPT4.0 | 6 | 47 |
| ChatGLM4 | 6 | 23 |
| ChatGLM4+Kb | 16 | 34 |
| ChatGLM3-6B | 18 | 49 |

**Table S3.** Self-positioning awareness.

| Large language models | Self-positioning awareness |
| --- | --- |
| ChatGPT4.0 | 27 |
| ChatGLM4 | 67 |
| ChatGLM4+Knowledge Base | 47 |
| ChatGLM3-6B | 10 |

**Table S4.** Medication safety.

| Large language models | Medication safety |
| --- | --- |
| ChatGPT4.0 | 87 |
| ChatGLM4 | 87 |
| ChatGLM4+Knowledge Base | 87 |
| ChatGLM3-6B | 87 |
